# Perceived stress and quality of life of pharmacy students in University of Ghana

**DOI:** 10.1186/s13104-017-2439-6

**Published:** 2017-03-02

**Authors:** Adomah Opoku-Acheampong, Irene A. Kretchy, Franklin Acheampong, Barima A. Afrane, Sharon Ashong, Bernice Tamakloe, Alexander K. Nyarko

**Affiliations:** 1 Department of Pharmacy Practice and Clinical Pharmacy, School of Pharmacy, College of Health Sciences, University of Ghana, Legon, Ghana; 2 Pharmacy Department, Korle Bu Teaching Hospital, Accra, Ghana; 3 Administration, School of Pharmacy, College of Health Sciences, University of Ghana, Legon, Ghana; 4 Department of Pharmacology and Toxicology, School of Pharmacy, College of Health Sciences, University of Ghana, Legon, Ghana

**Keywords:** Stress, Distress, Quality of life, Pharmacy, Students, Ghana

## Abstract

**Background:**

Stress among pharmacy students could greatly affect their learning activities and general well-being. It is therefore necessary to investigate how stress relates with the quality of life of students to maintain and/or improve their personal satisfaction and academic performance. A school-based longitudinal study was used to investigate the relationship between stress and quality of life of undergraduate pharmacy students. The 10-item perceived stress scale and the shorter version of the WHO quality of life scale were administered to the same participants at two time points i.e. Time 1 (4 weeks into the semester) and Time 2 (8 weeks afterwards). The correlations and differences between the study variables were tested using the Pearson’s coefficient and independent sample *t* test.

**Results:**

The mean stress scores were higher at Time 2 compared to Time 1 for the first and second years. However, there was no significant difference in stress for different year groups—Time 1 [F (3) = 0.410; p = 0.746] and Time 2 [F(3) = 0.909; p = 0.439]. Female students had higher stress scores at Time 2 compared to male students. The main stressors identified in the study were; large volume of material to be studied (88.2%), laboratory report writing (78.2%), constant pressure to maintain good grades (66.4%) and the lack of leisure time (46.4%). Even though most students employed positive stress management strategies such as time management (68.2%), other students resorted to emotional eating (9.1%) and alcohol/substance use (1.8%). At Time 2, perceived stress scores were significantly negatively correlated with social relationship (r = −0.40, p ≤ 0.0001), environmental health (r = −0.37, p ≤ 0.0001), physical health (r = −0.49, p ≤ 0.0001) and psychological health (r = −0.51, p ≤ 0.0001).

**Conclusion:**

The study reported significant correlations between stress and various domains of quality of life of undergraduate pharmacy students. It is thus necessary to institute some personal and institutional strategies to ameliorate the effect of stress on the quality of life of pharmacy students while encouraging the use of positive stress management strategies.

**Electronic supplementary material:**

The online version of this article (doi:10.1186/s13104-017-2439-6) contains supplementary material, which is available to authorized users.

## Background

Stress describes how a body reacts to external changes and is defined as “*the non*-*specific response of the body to any demand for change*” [1]. Stress can have both physical and psychological effects on individuals ranging from headaches, gastrointestinal discomfort, poor memory and difficulty with concentration [2].

Over the past few decades, stress among students has greatly increased with those in tertiary institutions being more liable [3–5]. The high level of stress has generally been attributed to the important academic and personal decisions these students usually make as they transit from adolescence into adulthood [6, 7].

Students of the health profession (medical, pharmacy, dental and nursing) have been reported to exhibit high levels of stress because of the nature of their educational process [8–10]. Pharmacy students demonstrate comparatively higher prevalence of stress than students of the other health professions which adversely affects their health and general quality of life [10]. Stress negatively affects the mental health of these students resulting in the development of stress-related disorders, low quality of life and poor academic performance [11–15]. In effect, some students may resort to certain strategies to alleviate stress. These interventions employed may affect quality of life affirmatively or negatively [10]. In view of this, some accreditation bodies for pharmacy education especially in the United States of America have recommended stress screening for their students to improve their performance academically [16].

In addition, previous studies on the stress among students of the health profession have predominantly been reported from the USA with emphasis on Doctor of Pharmacy students, as well as from other countries like the United Kingdom, United Arab Emirates, China, Malaysia and India [2, 6, 8, 11, 14, 17]. The relationship between high stress levels, low mental health-related quality of life and academic performance among pharmacy students have been reported [14, 17]. Yet, there is a dearth of information on stress and quality of life outcomes among pharmacy students in Ghana. To bridge this knowledge gap, the study sought to ascertain the relationship between stress and quality of life of pharmacy students, through the assessment of: (1) the experience and sources of stress among students at two time points of the semester, (2) the quality of life of students at two time points of the semester, (3) the correlation between perceived stress and quality of life of students, and (4) the stress management techniques used. The information obtained from the study will contribute to the literature on stress and quality of life of students in Ghana, which represents a sub-Saharan African country.

## Methods

### Study design and setting

This was a school-based longitudinal study using a paper survey to investigate the relationship between stress and quality of life of pharmacy students. Stress and quality of life were measured at two different periods within the semester at Time 1 (September 17 and 18, 2013) and Time 2 (November 5 and 6, 2013). The survey was conducted at a pharmacy school in Ghana.

### Participants

The entire population of one hundred and fifty-four (154) undergraduate students were eligible for the study and were recruited, representing 28, 31, 47 and 48 students in 4th, 3rd, 2nd, and the 1st years respectively. A return rate of 71.4% was obtained because 44 students opted out of the study at both occasions.

### Measures and procedure

A structured questionnaire was used in this study (Additional file 1) to gather information on socio-demographic characteristics, stress levels and quality of life of students. The extent to which participants viewed their lives as stressful, that is, overwhelming or unable to cope was measured using the 10-item perceived stress scale (Cronbach’s alpha of 0.82) [18–22]. The responses on the PSS vary from 0 to 4 and scored from 0 to 40 with higher scores indicating higher perceived stress. Items 4, 5, 7 and 8 on the PSS are positively worded and the scores were reversed [23]. The PSS is not a diagnostic instrument with no cut-off points hence comparisons were made within the sample.

The questionnaire also had a free-write section where the students were asked to list their most common stressors specific to pharmacy education and the coping techniques they employed. The various responses were content analyzed and presented descriptively.

In assessing the quality of life of participants, the shorter version of the WHO quality of life (WHOQOL-BREF) was used. It consists of twenty-six items (answered on a scale of 1–5) and measures four domains namely; physical health, psychological health, social relationships and environmental health with each domain having good psychometric properties [24]. Some facets incorporated into the physical health domain include activities of daily living, energy and fatigue, work capacity, sleep and rest. Some aspects of the psychological were negative feelings, positive feelings, self-esteem, bodily image and appearance. Personal relationships and social support were part of the social domain while financial resources, freedom, physical safety and security formed part of the facets under the environmental health. In scoring WHOQOL-BREF, items under a specific domain are separately scored with values ranging from 1 to 5. The sum up score for items in each domain was recorded as the raw domain score. Depending on the number of items present in a domain, the range of domain score varied. Per the guidelines, the raw domain scores were transformed to a 4–20 score which was comparable to the WHOQOL-100. The mean score of the items within each domain was used to calculate the domain score. This enabled the quality of life of participants under the various domains to be easily analyzed where higher scores denoted higher quality of life [25].

The questionnaires were administered in English to participants on two occasions within the semester. The first was 4 weeks after the resumption of school and 8 weeks after, to prevent the direct stressful impact of examinations from influencing the results [17]. The test retest reliability for the PSS after 6 weeks have been reliable [26].

The participants were given envelope sealed copies of the paper questionnaires in their lecture rooms. They responded in their own time and privacy and the sealed filled out questionnaires were collected the following day. The questionnaire did not have aspects that allowed for the personal identifiable information about the participants to ensure confidentiality. Additionally, the participants were given envelopes to contain the filled-out questionnaires. This was to ensure that reliability challenges with direct observational studies or the provision of socially desirable responses were minimized.

### Ethics

Approval to conduct the study was given by the research committee and school authorities before the commencement of the data collection. Taking part in this study was by choice and all participants willing to be part of the study gave informed written consent after the study objectives had been clearly understood by them. Each participant was assigned a code to ensure anonymity of participants and confidentiality of the information obtained.

### Data analysis

The data obtained were analyzed using Statistical Package for the Social Sciences (SPSS-version 20). The Kolmogorov–Smirnov test for normality was not significant (p = 0.712). Missing data were replaced with mean scores of the variables. Comparison and correlations between stress and quality of life of students at Time 1 and 2 were conducted using independent samples t-test and the Pearson correlation tests respectively. Responses on the most common stressors specific to pharmacy education and the coping techniques used were structured, coded and content analyzed. The codes were generated from a careful selection of excerpts of the responses and organized into categories. These have been presented descriptively as frequencies and percentages. A pilot study was conducted involving two students from each year group to assess the appropriateness of the study tool and the questions were clear to all of them.

## Results

Out of 154 eligible students, 110 participated in the study at both Times 1 and 2. Most of the respondents were males (64.5%) with most of them (70%) between the ages of 20 and 25. The students in the 1st, 2nd, 3rd, and 4th years constituted 22.7, 35.5, 17.3 and 24.5% of the study participants respectively. Table 1 shows the demographic characteristics of the participants.

**Table 1:** Socio-demographic information of respondents

Item	Category	Frequency	Percentage (%)
Gender	Male	71	64.5
	Female	39	35.5
Age (years)	19	32	29.1
	20–25	77	70.0
	>25	1	0.9
Year of study	1	25	22.7
	2	39	35.5
	3	19	17.3
	4	27	24.5

### Stress among students

The level of stress experienced by students was measured with the perceived stress scale (PSS) at Times 1 and 2 (Table 2). The results showed that stress scores were the highest in the 4th year although the mean score decreased at Time 2. The mean stress scores were higher at Time 2 compared to Time 1 for the first and second years. However, there was no significant difference in stress for different year groups—Time 1 [F (3) = 0.410; p = 0.746] and Time 2 [F(3) = 0.909; p = 0.439].

**Table 2:** Stress scores for year of study and gender

	Time 1	Time 2
	Mean (±SD)	Mean (±SD)
Year of study		
1	17.64 (7.199)	18.43 (7.555)
2	17.72 (5.862)	19.41 (5.919)
3	17.42 (5.60)	17.32 (5.447)
4	19.15 (6.311)	17.00 (6.481)
Gender		
Male	17.85 (6.239)	17.4 (5.193)
Female	18.29 (6.234)	19.55 (7.712)

No significant difference in stress for different year groups Time 1 [F(3) = 0.410; p = 0.746] and Time 2 [F(3) = 0.909; p = 0.439]

Unlike male students whose mean stress levels varied minimally [Time 1 (mean = 17.85 ± 6.239) and Time 2 (mean = 17.40 ± 5.193)], female students had higher stress scores during the semester [Time 1 (mean = 18.29 ± 6.234) and Time 2 (mean = 19.55 ± 7.712)].

From Table 3, the majority (93.6%) of participants indicated that pharmacy education was stressful. The distribution of what participants perceived as causes of stress (multiple responses) were large volume of material to be studied (88.2%), laboratory report writing (78.2%), constant pressure to maintain good grades (66.4%), and the lack of leisure time (46.4%) with the least cause of stress identified as poor quality of teaching (5.5%).

**Table 3:** Perceptions of stress, stressors and coping

Stress	Frequency	%
Perception of stress		
Pharmacy is stressful	103	93.6
Stress affects academic performance	74	67.3
Stress is manageable	99	90.0
Employ management strategies	97	88.2
Stressors		
Large study materials	97	88.2
Laboratory report writing	86	78.2
Pressure to maintain good grades	73	66.4
Lack of leisure time	51	46.4
Poor teaching quality	6	5.5
Management strategies		
Time management	75	68.2
Listening to music	64	58.2
Time with family/friends	57	51.8
Regular relaxation	56	50.9
Emotional eating	10	9.1
Alcohol and/or drug use	2	1.8

There were multiple responses

Although 67.3% of the students recognized that stress affected their academic performance, the majority (90%) of the participants perceived their stresses as manageable. Stress management strategies were employed by 88.2% of the respondents which was mainly positive. These included time management (68.2%), regular relaxation (50.9), listening to music (58.2%) and spending time with families and/or friends (51.8%). However, some respondents used strategies which could negatively affect their health such as, emotional eating (9.1%) and alcohol/substance abuse (1.9%).

### Quality of life of students

The mean scores for quality of life are summarized in Table 4. Generally, participants in each year of study reported higher quality of life scores at Time 2 compared to Time 1 and the participants in third year reported the highest mean quality of life. There were no significant differences in quality of life for different year groups at Time 1 [F(3) = 0.409; p = 0.747] and Time 2 [F(3) = 1.316; p = 0.273]. Similarly, the mean quality of life scores for both males and females at Time 2 were higher than that of the scores at Time 1. However, the difference in quality of life between males and females was insignificant at Time 1 [F (1) = 1.951; p = 0.165] and Time 2 [F (1) = 0.018; p = 0.893].

**Table 4:** Quality of life scores for year of study and gender

Quality of life	Time 1	Time 2
	Mean (±SD)	Mean (±SD)
Year of study		
1	77.50 (12.938)	80.43 (17.959)
2	75.90 (14.639)	78.21 (11.669)
3	80.00 (11.055)	85.26 (6.118)
4	77.41 (12.888)	81.48 (12.949)
Gender		
Male	76.06 (13.678)	80.61 (11.077)
Female	79.74 (11.965)	80.95 (15.588)

No significant differences in quality of life for different year groups Time 1 [F(3) = 0.409; p = 0.747] and Time 2 [F(3) = 1.316; p = 0.273] and gender Time 1 [F(1) = 1.951; p = 0.165] and Time 2 [F(1) = 0.018; p = 0.893]

### Relationship between stress and quality of life

There were significant correlations between the overall stress and quality of life at both Time 1 (r = −0.383; p < 0.001) and Time 2 (r = −0.487; p < 0.001). The overall perceived stress and quality of life are presented in Table 5 and the correlations between stress and each quality of life domain are summarized in Table 6. Except for social relationship (r = 0.11, p = 0.251) with stress, all other dimensions had a significant negative correlation with perceived stress at Time 1 i.e. environmental health (r = −0.37, p ≤ 0.0001), physical health (r = −0.45, p ≤ 0.0001) and psychological health (r = −0.55, p ≤ 0.0001). However, at Time 2, all the dimensions had a negative correlation with stress i.e. social relationship (r = −0.40, p ≤ 0.0001), environmental health (r = −0.37, p ≤ 0.0001), physical health (r = −0.49, p ≤ 0.0001) and psychological health (r = −0.51, p ≤ 0.0001).

**Table 5:** Overall perceived stress and quality of life (QoL)

Variable	Time 1			Time 2			t (j−k)	df	p-value
	Mean (j)	SD	Min–max	Mean (k)	SD	Min–max			
Stress	18.06	6.212	3–33	18.24	6.356	5–40	−0.214	106	0.831
QoL	77.34	13.169	30–100	80.74	12.949	20–100	−1.868	107	0.065

Significant correlations between overall stress and QoL at both Time 1 (r = −0.383; p < 0.001) and Time 2 (r = −0.487; p < 0.001)

**Table 6:** Correlation between stress and different quality of life domains

QoL dimension	Time 1		Time 2	
	r value	p-value	r value	p-value
Social relationship	0.11	0.251	−0.40	≤0.0001
Environmental health	−0.37	≤0.0001	−0.37	≤0.0001
Physical health	−0.45	≤0.0001	−0.49	≤0.0001
Psychological health	−0.55	≤0.0001	−0.51	≤0.0001

## Discussion

This study focused on the experience of stress and quality of life of undergraduate pharmacy students. Similar to observations made by Gallagher et al. [11], stress was reported among the participants though the difference in stress scores for various classes was insignificant. Unlike findings from other studies where statistically significant differences in stress were observed for various years of study [27, 28], our findings are corroborated in a study among undergraduate pharmacy students where no significant differences were observed [17]. For those in first and second years however, the stress levels increased by Time 2. This could be attributed to the fact that by Time 2 academic work would have peaked and students were required to submit assignments and regular laboratory reports while preparing for their mid and end of semester examinations [17]. Additionally, the first-year students may probably be experiencing challenges of adapting to their new environment while previous studies have also indicated that the second-year curriculum was heaving because of the transition from studies in basic sciences to mostly pharmaceutical science-related courses in preparation for their pre-clinical studies [12].

In this study, the percentage of female students (35.5%) was lower than the percentage of male students (64.5%) and the female students reported higher mean scores of perceived stress than males [29–31]. Yet, this differences between male and female students in their perception of stress during their study was not significant [32, 33]. This result probably indicates that using participants from a single study site exposed them to similar perceptions of stress and stressors. While no significant relationship has been observed [17] a significant association between gender and perceived stress has been reported [14].

The current study showed that most students perceived pharmacy education as stressful and could affect their academic performance. The participants identified large volume of material required to be studied as the most common stressor. The Bachelor of Pharmacy programme undertaken at the pharmacy school was a 4-year programme comprised of both theoretical and laboratory practical courses in the sciences such as pharmaceutical chemistry, pharmaceutics, pharmaceutical microbiology, pharmacognosy, pharmacology, clinical pharmacy as well as social and behavioral pharmacy [34, 35]. These courses involved large volumes of material to be studied. Laboratory report writing is a requirement for the students after each practical laboratory exercise in the pharmaceutical sciences. This report writing gives a fair idea of how well the student understood the laboratory work. It involves thorough research in order to discuss the results obtained. Within a week, students may have about four or five laboratory works to report on, with each work to be submitted 48 h after the end of the laboratory work. This may have accounted for the related stress.

Most students are stressed due to high standards they set for themselves and/or pressure from parents to perform well in school [36]. The study participants may be exerting themselves to be academic achievers even at the expense of their required amount of sleep and previous studies have indicated that students mostly study at dawn, enjoying few hours of sleep each night [37, 38]. Inadequate relaxation and socialization is reported to negatively affect the quality of life of individuals especially their psychological well-being and this may probably explain why there was the lack of leisure time among participants [39].

Students were noted to employ both positive and negative stress management strategies in accordance with a study conducted by Al-Dubai et al. [15]. Time management was the strategy employed by respondents to reduce their stress levels. Managing one’s time is an effective stress management strategy as it helps in achieving goals as planned without excessively stressing oneself. Some participants opted for music with notable soothing effect on the body and mind [40, 41]. Time with family and friends when stressed also enabled participants to express their negative emotions with their significant others [31]. While the use of alcohol and other substances as well as emotional eating were noted, these findings emphasize the need to address unhealthy stress management strategies among students. Some forms of extra curricula activities for students could be initiated by the school to encourage leisure and relaxation.

There was no significant difference in quality of life measures for male and female students as well as that for the various years of study though the second year students generally reported lower quality of life scores [42]. Similarly, the correlations between overall stress and overall quality of life at both Times 1 and 2 were not significant. However, the relationship between stress and each specific domain of the quality of life of participants during the semester was negative and significant. The findings that stress decreased some domains of quality of life is consistent with previous studies [12, 14, 29, 43]. Two of these studies examined the effects of stress on quality of life among PharmD students and reported that higher levels of stress negatively correlated with the mental domain of quality of life [14, 29]. Per Awadh et al. [43], there was a negative correlation between perceived stress levels and the physical component of quality of life among MPharm students. This study however, reported a negative relationship between stress and all measured aspects of quality of life (i.e. environmental, physical, psychological and social) which is consistent with a study conducted among both undergraduate and graduate students [7]. This observation could be partly due to the difference in curricular between the BPharm, MPharm and Pharm D programmes or partly attributed to the fact that the students, particularly, undergraduates, had less time to maintain or improve their social relationship and other domains of quality of life. Contrary to the pass/fail grading system, the BPharm undergraduate programme has the A, B, C, D system of grading with strict cut-off points. In line with a previous study, the strict cut-off grading system has been noted to significantly reduce the general well-being of medical students [44].

Information on stress and quality of life among pharmacy students in Ghana is limited and this study has provided some information to bridge this knowledge gap. The study however acknowledges some limitations. First, the study was conducted in one out of the three pharmacy schools located in Ghana. The interplay between stress and quality of life may be different in the other institutions and this can be further studied because no available data has been published. Second, the study did not objectively report on how stress and quality of life translated into academic performance using the grade points. The association between these variables merit further investigation. Finally, potential non-response bias was not addressed which led to about 28% non-response rate among the study participants.

## Conclusions

The study reported significant negative correlations between stress and the various domains of quality of life of undergraduate pharmacy students. It is thus necessary to institute some personal and institutional strategies to ameliorate the effect of stress on the quality of life of pharmacy students while encouraging the use of positive stress management strategies.

## Additional file

**Additional file 1.** Questionnaire.
