# Supplementary material for: Perceived stress and quality of life of pharmacy students in University of Ghana
Source: BMC Res Notes. 2017 Mar 2;10:115. doi: 10.1186/s13104-017-2439-6 (PMC5335855; doi:10.1186/s13104-017-2439-6)
Supplement: Supplementary file 1 — Additional file 1. Questionnaire. [file 13104_2017_2439_MOESM1_ESM.docx]

**UNIVERSITY OF GHANA SCHOOL OF PHARMACY**

**DEPARTMENT OF PHARMACY PRACTICE AND CLINICAL PHARMACY**

**PART I**

1. Gender/sex …………….. (a) Male (b) Female
2. Age ……………………... (a) 19yrs or below (b) 20 – 25yrs (c) above 25yrs
3. Present level …………… (a) 100 (b)200 (c) 300 (d) 400
4. Marital status …………. (a) Single (b) In a relationship (c) Married (d) Others

**PART II**

This is a measure of the degree to which you are experiencing stress in your various life situations. For each item choose the number that best describes you by circling one of the five numbers to the right of the statement according to the following scale:

0=never 1=almost never 2=sometimes 3=fairly often 4=very often

1. In the last month, how often have you been upset because of something that happened unexpectedly? 0 1 2 3 4
2. In the last month, how often have you felt that you were unable to control the important things in your life? 0 1 2 3 4
3. In the last month, how often have you felt nervous and “stressed”? 0 1 2 3 4
4. In the last month, how often have you felt confident about your ability to handle your personal problems? 0 1 2 3 4
5. In the last month, how often have you felt that things were going your way?

0 1 2 3 4

1. In the last month, how often have you found that you could not cope with all the things you had to do? 0 1 2 3 4
2. In the last month, how often have you been able to control irritations in your life?

0 1 2 3 4

1. In the last month, how often have you felt that you were on top of things? 0 1 2 3 4
2. In the last month, how often have you been angered because of things that were outside of your control? 0 1 2 3 4
3. In the last month, how often have you felt difficulties were piling up so high that you could not overcome them? 0 1 2 3 4

**PART III**

1. Was it your personal choice to offer Pharmacy as a course? (a) Yes (b) No
2. Do you feel Pharmacy education is ‘stressful’? (a) Yes, it is (b) No, it isn’t
3. If yes, what are some of your identified stressors (causes of stress)?

Please indicate ALL stressor(s). a)………………………………………………………………………………………b)………………………………………………………………………………………c)………………………………………………………………………………………d)………………………………………………………………………………………e)……………………………………………………………………………………….f)……………………………………………………………………………………….g)………………………………………………………………………………………h)……………………………………………………………………………………….i)……………………………………………………………………………………….. j)………………………………………………………………………………………………………………………………………………………………………………………………………………………………………………………………………………………………………………………………………………………………………

1. Does the stress you are encountering affect your academic performance? (a) Yes (b) No

Explain your answer. .…………………………………………………………………………………………………………………………………………………………………………………………………………………………………………………………………………………………………………………………………………………………………………………………………………

1. Do you think stress among Pharmacy students can be managed? (a) Yes (b) No
2. Do you employ any strategies to help you reduce/manage stress? (a) Yes (b) No
3. If yes, which of these strategies do you employ?
4. Time management
5. Regular relaxation
6. Regular exercise
7. Smoking
8. Emotional eating
9. Alcohol and drug use
10. Listening to music
11. Time with family and/or friends

Any other strategy/strategies you employ not stated above? …………………………………………………………………………………………………………………………………………………………………………………………………………………………………………………………………………………………………………………………………………………………………………………………………………………………………………………………………………………………………………

1. Suggest ways you think stress among University of Ghana School of Pharmacy students could be reduced or managed. …………………………………………………………………………………………………………………………………………………………………………………………………………………………………………………………………………………………………………………………………………………………………………………………………………

**PART IV**

Instructions

This part asks how you feel about your quality of life, health and other areas of your life. Please answer all the questions. If you are unsure about which response to give to a question, please choose the ONE that appears most appropriate. This can often be your first response. Please keep in mind your standards, hopes, pleasures and concerns. We ask that you think about your life in the last two weeks. For example, thinking about the last two weeks, a question might ask:

Do you get the kind of support from others that you need?

Not at all Not much Moderately A great Deal Completely

1 2 3 4 5

You should choose the number that best fits how much support you got from others over the last two weeks. So you would circle the number 4 if you got a great deal of support from others but you would circle the number 1 if you did not get any of the support that you needed from others in the last two weeks.

Please read each question, assess your feelings, and choose the number on the scale for each question that gives the best answer for you.

|  | Very Poor | Poor | Neither Poor nor Good | Good | Very Good |
| --- | --- | --- | --- | --- | --- |
| 1 How would you rate your quality of life? |  |  |  |  |  |

|  | Very Dissatisfied | Dissatisfied | Neither Satisfied nor Dissatisfied | Satisfied | . Very Satisfied |
| --- | --- | --- | --- | --- | --- |
| 2 How satisfied are you with your health? |  |  |  |  |  |

The following questions ask about how much you have experienced certain things in the last two weeks.

|  | Not At All | A Little | A Moderate Amount | Very Much | An Extreme Amount |
| --- | --- | --- | --- | --- | --- |
| 3 How much do you feel that pain prevents you from doing what you need to do? |  |  |  |  |  |
| 4 How much do you need medical treatment to function in your daily life? |  |  |  |  |  |
| 5 How much do you enjoy life? |  |  |  |  |  |

|  | Not At All | A Little | A Moderate Amount | Very Much | Extremely |
| --- | --- | --- | --- | --- | --- |
| 6 To what extent do you feel life to be meaningful? |  |  |  |  |  |
| 7 How well are you able to concentrate? |  |  |  |  |  |
| 8 How safe do you feel in your daily life? |  |  |  |  |  |
| 9 How healthy is your physical environment? |  |  |  |  |  |

The following questions ask about how completely you experience or were able to do certain things in the last two weeks.

|  | Not At All | A Little | Moderately | Mostly | Completely |
| --- | --- | --- | --- | --- | --- |
| 10 Do you have enough energy for everyday life? |  |  |  |  |  |
| 11 Are you able to accept your bodily appearance? |  |  |  |  |  |
| 12 To what extent do you have enough money to meet your needs? |  |  |  |  |  |
| 13 How available to you is the information that you need in your day-to-day life? |  |  |  |  |  |
| 14 To what extent do you have the opportunity for leisure activities? |  |  |  |  |  |

The following questions ask you to say how good or satisfied you have felt about various aspects of your life over the last two weeks.

|  | Very Poor | Poor | Neither Poor nor Good | Good | Very Good |
| --- | --- | --- | --- | --- | --- |
| 15 How well are you able to get around? |  |  |  |  |  |

|  | Very Dissatisfied | Dissatisfied | Neither Satisfied nor Dissatisfied | Satisfied | . Very Satisfied |
| --- | --- | --- | --- | --- | --- |
| 16 How satisfied are you with your sleep? |  |  |  |  |  |
| 17 How satisfied are you with your ability to perform daily living activities? |  |  |  |  |  |
| 18 How satisfied are you with your capacity for work? |  |  |  |  |  |
| 19 How satisfied are you with yourself? |  |  |  |  |  |
| 20 How satisfied are you with your personal relationships? |  |  |  |  |  |
| 21 How satisfied are you with your sex life? |  |  |  |  |  |
| 22 How satisfied are you with the support you get from your friends? |  |  |  |  |  |
| 23 How satisfied are you with the conditions of your living place? |  |  |  |  |  |
| 24 How satisfied are you with your access to health services? |  |  |  |  |  |
| 25 How satisfied are you with your transport? |  |  |  |  |  |

The following question refers to how often you have felt or experienced certain things in the last two weeks.

|  | Never | Seldom | Quite Often | Very Often | Always |
| --- | --- | --- | --- | --- | --- |
| 26 How often do you have negative feelings, such as blue mood, despair, anxiety, depression? |  |  |  |  |  |

THANK-YOU FOR YOUR HELP
